# Investigation of pixel scale calibration on the Elekta iView electronic portal imager

**DOI:** 10.1002/acm2.13339

**Published:** 2021-07-11

**Authors:** Craig Norvill, Simon Goodall

**Affiliations:** ^1^ Genesis Care Perth Australia; ^2^ School of Physics, Mathematics, and Computing Faculty of Engineering and Mathematical Sciences University of Western Australia, Crawley Western Australia Australia

**Keywords:** Elekta, iView, pixel scale

## Abstract

This study investigated the variation in electronic portal imager pixel scale at the isocenter plane for Elekta Agility linear accelerators. An in‐house MATLAB script was written to process and calculate the pixel scale based on a metal calibration plate supplied by Elekta. Eight pixel plates were compared and found to have manufacturing tolerances within 0.1 mm of nominal dimensions. The impact of these variations on pixel scale factor was negligible, and plates could be used interchangeably. Uncertainties from other parameters such as source‐to‐surface distance and user variability summed to a combined uncertainty of 0.0003 mm/pixel, compared to a pixel scale range of 0.003 mm/pixel measured across 10 machines. Most of the inter‐machine variation was shown to be attributable to differences in source‐to‐panel distance. Other factors such as focal spot size and shape, electronic portal imager manufacturing consistency, panel sag, and setup errors may account for the residual variation. Individual characterization of machine and imaging panel pixel scale factors is important to ensure accurate geometric information is derived from electronic portal images, which is critical where the portal imager is used for multi‐leaf collimator calibration or other clinical tasks.

## 1 INTRODUCTION

Electronic portal imaging devices (EPIDs) have become an essential component on modern radiotherapy linear accelerators and are integral to patient setup, registration, and position correction workflow. With the increasing use of volumetric arc therapy (VMAT) techniques and kilovoltage cone beam CT (CBCT), the main function of the megavoltage EPID panel in many departments is for use as a quality assurance (QA) tool. A number of publications describe the use of megavoltage EPID panels for tests such as MLC position calibration, dose‐output constancy, beam flatness/symmetry, radiation field size, and kilovoltage (KV) to megavoltage (MV) isocenter calibration/verification.^1^, ^2^, ^3^, ^4^, ^5^, ^6^

The EPID is typically located at an extended source‐to‐panel distance, however, for many tests there is a need to specify the radiation field at the machine isocenter plane, typically 100 cm source‐to‐axis (SAD). To achieve this, image data defined in EPID pixels needs to be converted to distance (e.g., millimeters) at isocenter. The EPID has fixed pixel dimensions, so a knowledge of the EPID pixel size, SAD, and source‐to‐panel (SPD) distances allow for conversion of EPID images in pixels at SPD to millimeters at the SAD.

The Elekta EPID is an amorphous silicon detector manufactured by Perkin Elmer, consisting of 1024 × 1024 16‐bit pixels.^7^ The panel has a dimensions of 41 cm × 41 cm and for the Elekta Agility model linear accelerator (Elekta, Stockholm, Sweden) it is nominally positioned at 160 cm SPD, giving an approximate 26 cm × 26 cm maximum extent at the isocenter plane. Electrons produced in the aluminium and copper plates are converted to visible light by a scintillator plate and are then converted to an electrical signal by a photodiode array. Image data are processed and digitized by a frame‐grabber, giving a near real‐time image. Custom software (Elekta iView) controls the image acquisition and processing and provides a user interface to the hardware.

**FIGURE 1:**
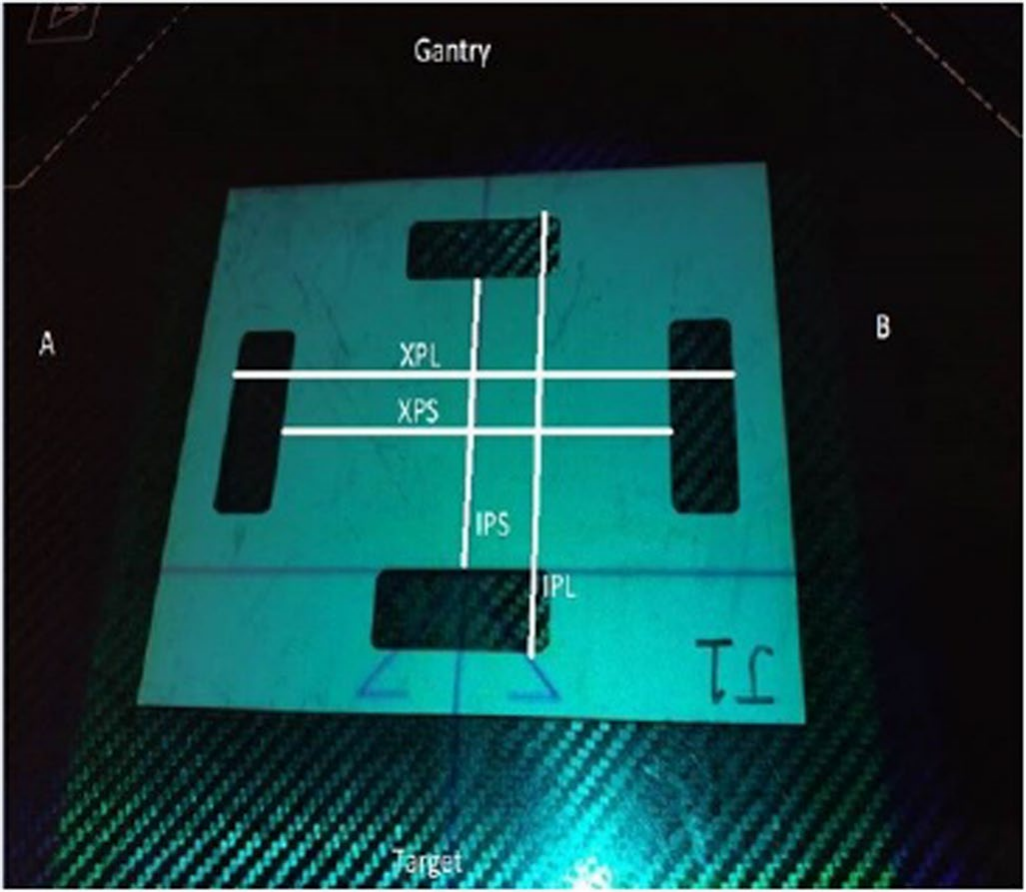
Elekta pixel calibration plate as oriented on treatment table. Dimensions used to determine pixel scale factor are illustrated

**FIGURE 2:**
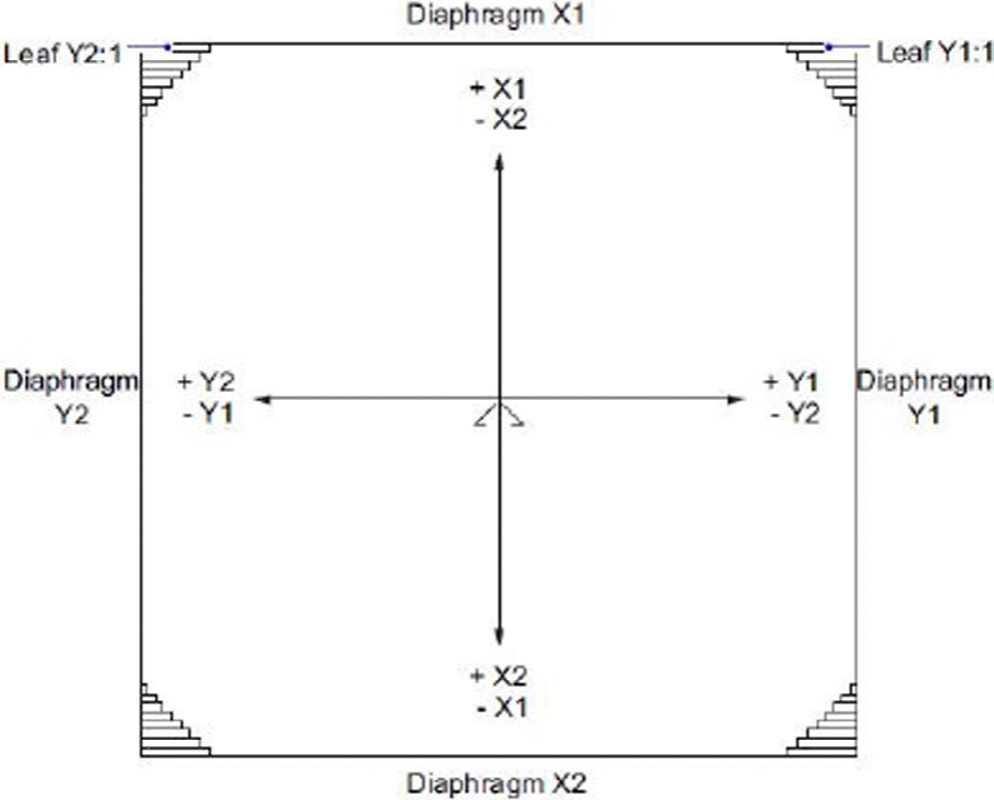
IEC60601 co‐ordinate convention, adapted from Elekta Digital Linear Accelerator Customer Acceptance Test^10^

For the Elekta Agility linear accelerator, pixel scale factor is measured during the automated multi‐leaf collimator beam limiting device (BLD) calibration workflow. This is achieved using a 200 mm × 155 mm metal plate, (Figure 1) which has apertures of nominal fixed dimensions. The co‐ordinate reference frame used in the Elekta BLD workflow is defined by IEC 60601.^8^ This is illustrated in Figure 2, and is used as the reference co‐ordinate frame for this paper.

To determine a pixel scale factor, the BLD workflow sets a field size of X1 = 12 cm, X2 = 4 cm, Y1 (MLC) = 12 cm, and Y2 (MLC) = 12 cm. Gantry and collimator are set to zero degrees, and a source‐to‐table surface (SSD) of 100 cm is set by the user. The calibration plate is aligned to the optical cross‐wires and an exposure acquired. Pixel scale factor is calculated by the BLD algorithm in cross‐plane (A–B) and in‐plane (Gantry‐Target) directions, according to Equation (1). The algorithm used to extract aperture pixel dimensions from the image is not articulated by Elekta in user manuals available to the authors. As no user input is required for the plate dimensions, it is assumed that the algorithm uses nominal plate aperture dimensions.^9^

(1) $$PixScale\left(\frac{\mathrm{mm}}{\mathrm{pixel}}\right)=\frac{Apeturedimensionatisocenterplane\left(\mathrm{mm}\right)}{AperturedimensionatEPIDplane\left(\mathrm{pixels}\right)}$$

The calculated pixel scale factor is subsequently used in the BLD workflow position calculation and hence the calibration of multi‐leaf collimator positions over the range of measured leaf travel. It was noted that variation in pixel scale factor across the Elekta Agility linear accelerators in our institution was significant enough to have a clinically relevant impact on MLC calibration should the measured pixel factor be inaccurate. Furthermore, the independent MLC QA system used in our institution (AQUA) was also EPID based and required input of the pixel scale factor. To be unbiased, this value should be derived independently of the Elekta BLD workflow. The purpose of this paper is to compare an independent in house algorithm to the Elekta BLD pixel scale workflow, evaluate factors influencing the accuracy of measured pixel scale factor such as pixel plate dimensional accuracy and plate setup accuracy, and to quantify the variation in measured pixel scale factor across multiple Elekta Agility linear accelerators.

## 2 MATTERIALS AND METHODS

### 2.1 Pixel calibration plate dimensions

The Elekta pixel calibration plate and associated dimensions measured in this study are shown in Figure 1. Dimensions were defined as cross‐plane long (XPL), cross‐plane short (XPS), in‐plane long (IPL), and in‐plane short (IPS). These dimensions have nominal lengths of 170, 130, 125, and 85 mm, respectively. Eight calibration plates were measured in the same session using digital Vernier callipers with a precision of 0.01 mm. Each dimension was measured three times, at positions 1/4, 1/2, and 3/4 along the aperture width. The XPL dimension was too large for the callipers and, therefore, measured using a steel rule to an estimated precision of 0.25 mm. Mean, standard deviation, and range were recorded for each dimension of each pixel plate.

### 2.2 Pixel scale factor variation with calibration plate

Portal images of each of the eight calibration plates were acquired on machine “A” in a single session. Gantry and collimator were set to 0º, and the source‐to‐table distance was set to 100 cm. A 6 MV stored beam was configured with dimensions of Y1 = 12 cm, Y2 = 12 cm, X1 = 12 cm, and X2 = 4 cm. Beam monitor units were set to 20.

Each calibration plate was aligned to the crosswire markings as illustrated in Figure 1. In this orientation, the cross‐plane direction (A–B) contains the XPL and XPS dimensions, while the in‐plane direction (G–T) contains the IPL and IPS dimensions. Ten images were acquired for each plate, with the measured dimensions being the average of the 10 images.

**FIGURE 3:**
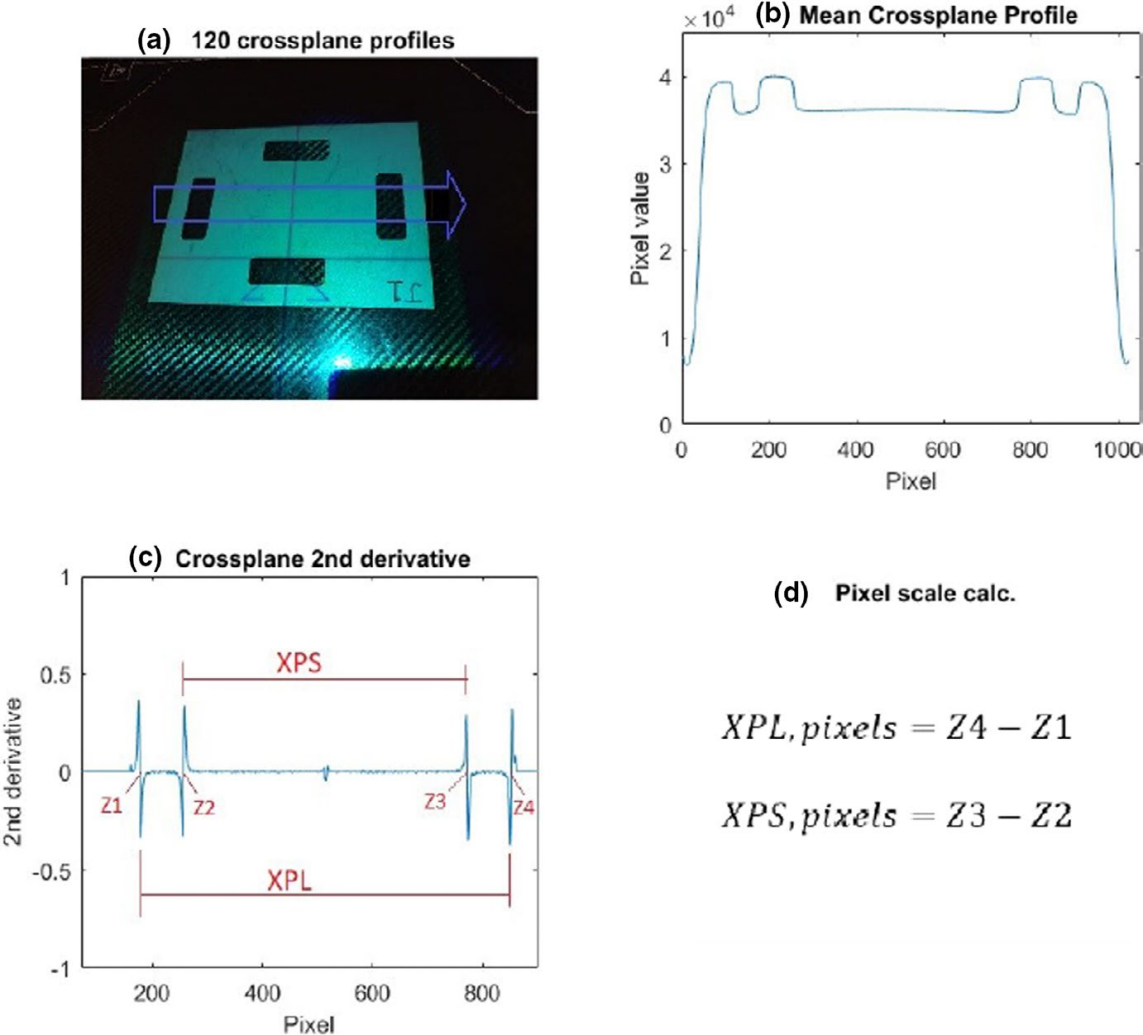
Workflow for MATLAB script to determine cross‐plane pixel scale, (a) determine 120 cross‐plane profiles across pixel plate cut‐out, (b) calculate mean and filter, (c) take second derivative, and (d) calculate dimensions cross‐plane long and cross‐plane short in pixels

Images were processed using an in‐house MATLAB (MathWorks, Natick, MA) script, the workflow of which is described in Figure 3. For the cross‐plane and in‐plane directions the average of 120 profiles (30 mm) were taken, and the resulting mean profile was filtered using a 5 pixel median smoothing function. The second derivative of the smoothed profile was calculated, and the four points where the function intercepted the zero‐axis was recorded. These points correspond to the four edges on the internal pixel plate cut‐outs. The dimensions as shown in Figure 3 were then calculated in terms of pixels, and the pixel scale factor was calculated using nominal plate dimensions. The script was then run using the calliper measured plate aperture dimensions in place of the nominal values. Variation in pixel scale factor across plates with nominal and measured dimensions was compared against theoretically expected differences.

### 2.3 Influence of source‐to‐surface and source‐to‐panel distance

The intent of the pixel scale factor is to define the EPID scale at the machine isocenter (100 cm source‐to‐surface) plane. For fixed plate dimensions and source‐to‐panel distance, the magnification factor (SPD/SSD) will decrease with increasing SSD, or in other terms with increasing SSD the number of pixels measured at the panel decreases. The relationship of pixel scale factor to change in SSD is described in Equation (2).

(2) $$PixScale_{SSD_{2}}=PixScale_{SSD_{1}}\left(\frac{SSD_{2}}{SSD_{1}}\right)$$

Pixel plate “A” was setup on machine “A” with an SSD distance of 100 cm SSD, and images acquired as described previously. The SSD was varied between 99.5 cm and 100.5 cm in 0.1 cm intervals using a steel rule taped to a solid block, and using the horizontal wall localizing room lasers as a datum. For each SSD, three images were acquired and averaged.

The mean measured pixel scale factor was calculated and plotted as a function of SSD. The measured plot was compared to the theoretical change in pixel scale expected with SSD change.

Isocenter plane to EPID vertical distance (IPD) was measured by removing the iView EPID cover and measuring from the top surface of the EPID metal plate to the isocenter (coronal plane laser) using a meter steel rule. As part of routine QA procedures, all coronal lasers were previously validated to be within 0.5 mm of MV isocenter. Measurements were taken at the center and at the four corners of the EPID. Corner positions were defined as Gun‐A, Gun‐B, Target‐A, and Target‐B, where A‐Side is to the left of Gantry when viewing from foot of treatment table. SPD was calculated by adding 1000 mm to the IPD measurement.

### 2.4 Inter‐machine variation

Pixel scale factor was measured across 10 Elekta Agility linear accelerators using a single pixel plate, “A.” For each machine, 10 images were acquired, and processed as described previously. Variation in pixel scale factor was evaluated across all machines for in‐plane and cross‐plane directions. Reference Elekta BLD pixel scale factors, acquired for each machine at various dates over the preceding 12 months were compared to the MATLAB calculated values.

## 3 RESULTS

### 3.1 Pixel calibration plate dimensions

**FIGURE 4:**
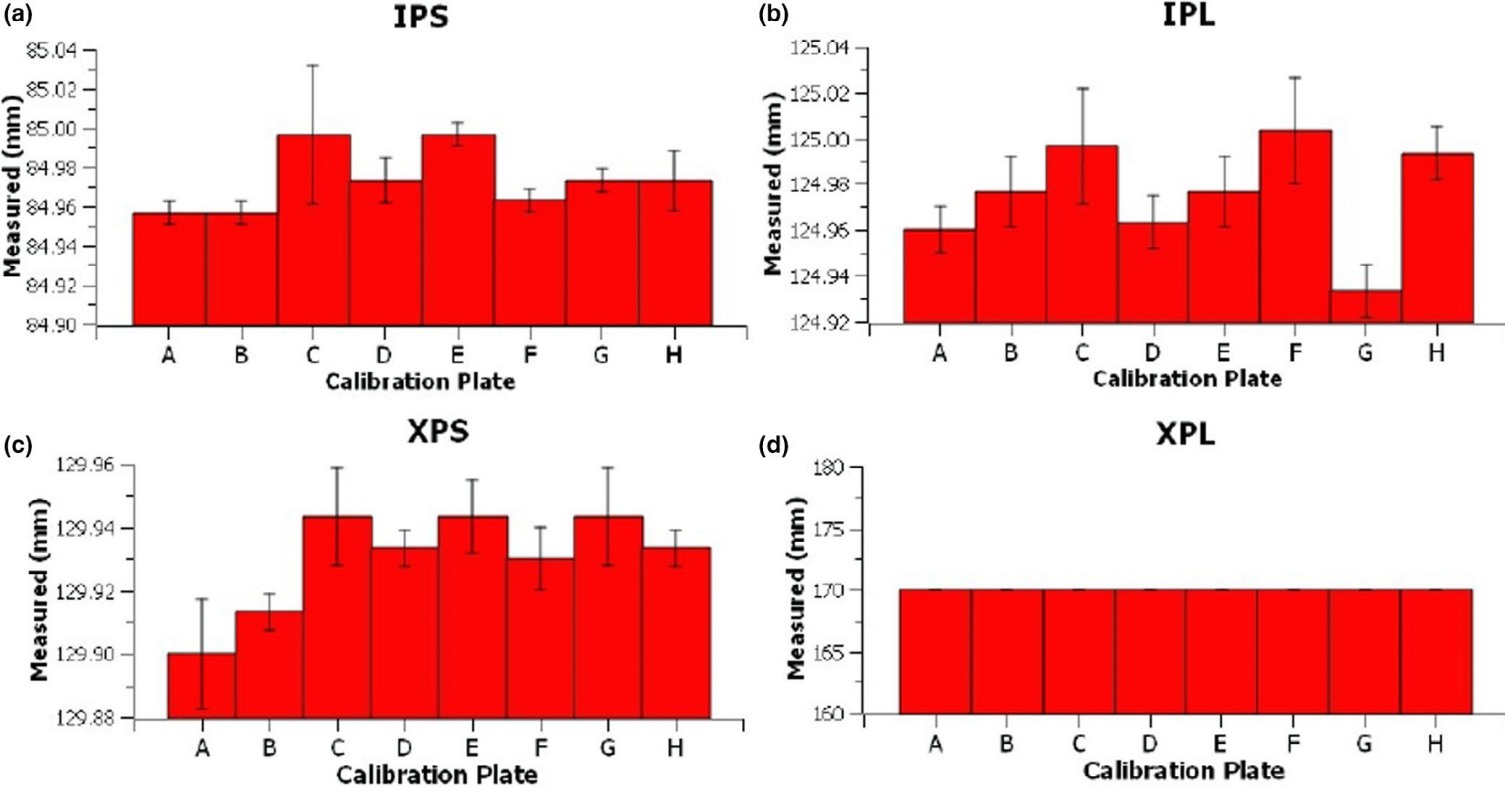
Measured calibration plate dimensions for dimension (a) in‐plane short, (b) in‐plane long, (c) cross‐plane short, and (d) cross‐plane long. Error bars represent the pooled standard deviation for all Vernier measurements

Vernier measured pixel plate dimensions for plates A–H are given in Figure 4. Pixel calibration plate dimensions matched the nominal values within −0.07 ± 0.02 mm, −0.02 ± 0.02 mm, and −0.03 ± 0.02 mm for the XPS, IPL, and IPS dimensions. Measurement dimension range for all eight calibration plates varied between 0.04 and 0.07 mm, with all plate dimensions within 0.1 mm of the nominal value. These measurements confirmed a reproducible manufacturing of the pixel plates. No variation in the XPL dimension was measured, which is consistent with the accuracy of the other dimensions, and resolution allowed by the steel rule.

### 3.2 Pixel scale factor variation with calibration plate

**FIGURE 5:**
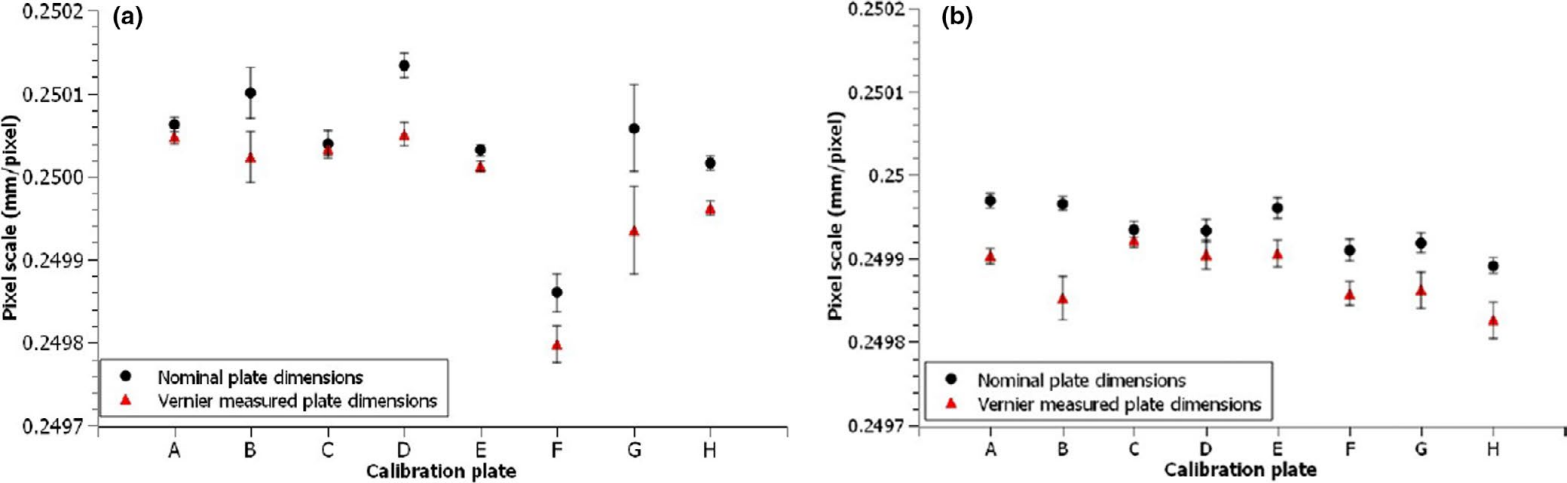
Measured pixel scale factor for eight calibration plates, using nominal and measured pixel scale factor for (a) in‐plane direction and (b) cross‐plane direction. Error bars indicate one standard deviation (n = 10 images per plate)

Figure 5 shows the average MATLAB calculated pixel scale factor (n = 10 images per plate) measured for plates A–H in both the in‐plane and cross‐plane directions. Error bars indicate one standard deviation. Results are presented using nominal plate dimensions and the Vernier measured dimensions. For the nominal plate dimensions, results ranged between 0.2498 and 0.2502 mm/pixel, with a mean of 0.2500. Use of Vernier measured rather than nominal plate dimensions gave a systematically lower pixel scale factor, which was expected given the Vernier measured plate dimensions were all generally smaller than the nominal dimensions. Differences were typically less than 0.0001 mm/pixel with a maximum difference of 0.00012 mm/pixel. With the exception of pixel plate F, cross‐plane direction pixel scale factor was smaller than the in‐plane value, with a mean difference of 0.0001 mm/pixel (Range −0.00005 to 0.0002). This suggests a small difference in the EPID pixel size for the cross‐plane relative to in‐plane direction.

### 3.3 Influence of source‐to‐surface and source‐to‐panel distance

**FIGURE 6:**
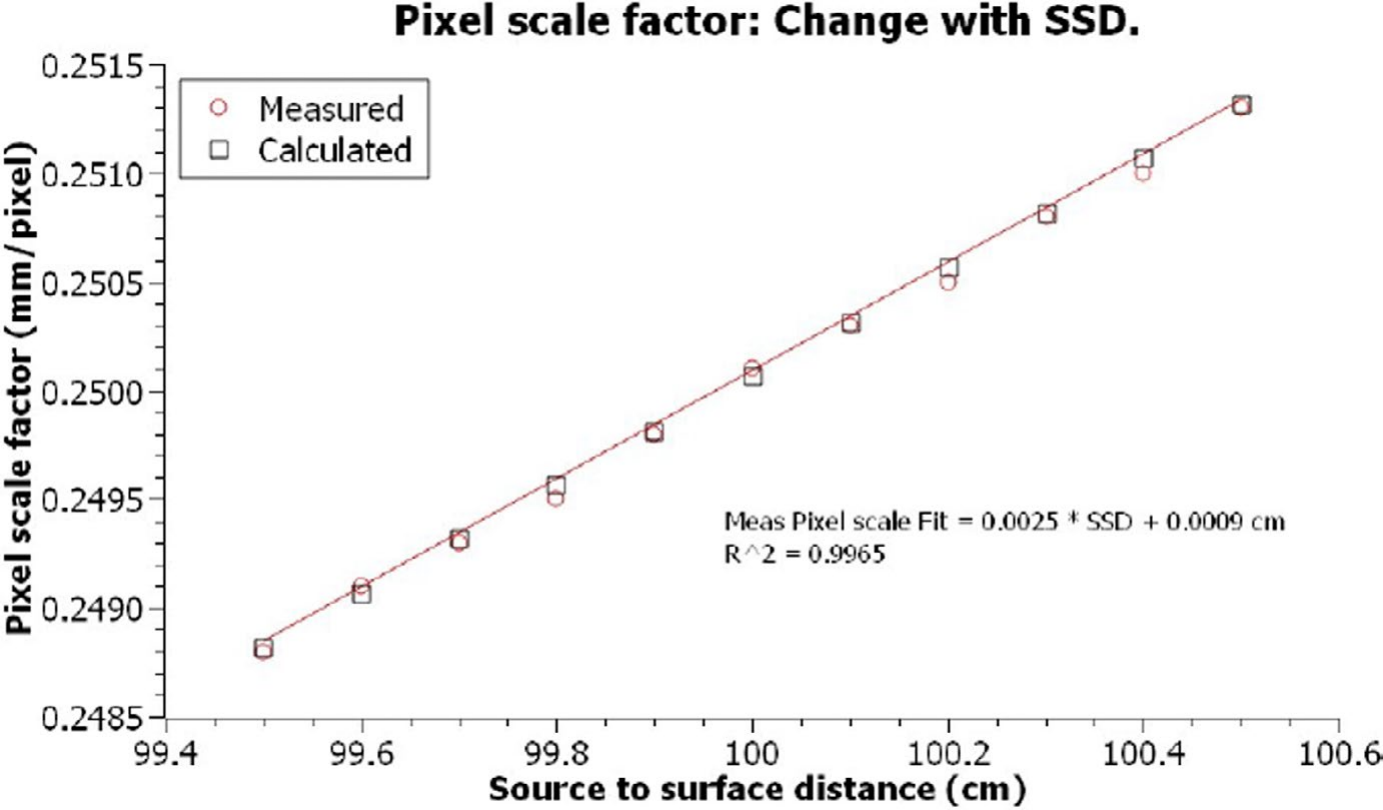
Variation in pixel scale factor with source‐to‐surface distance

Variation in pixel scale with source‐to‐surface distance variation is given in Figure 6. Error bars equivalent to one standard deviation were plotted but not visible at the graph resolution. The data follows a linear trend, and regression fit shows that for each millimeter variation in SSD, the pixel scale factor varied by 0.00025 mm/pixel (R^2^ = 0.9965). The theoretical data are also plotted and was normalized to the measured pixel scale at 100 cm SSD. Measured data aligned nearly exactly with theoretical curve. Any minor deviations were likely a result of the precision with which the SSD could be set (~0.25 mm) using a steel rule and room localizing laser.

Isocenter plane to EPID vertical distance (IPD) measurements for each linear accelerator are presented in Table 1, measured at the center and at the four corners of the panel. Across all machines, IPD measured at center of the panel ranged between 579 and 600 mm, with a mean of 591 mm (SD 6 mm). All panels exhibited a sag at the target end of the panel relative to gantry end. From the corner measurements in Table 1, the target end across all machines was on average 4 mm (SD 2 mm) lower than the gun end. Maximum sag was reported for machine “D” with a 6 mm difference. All panels were horizontal in the A–B direction, with average variation less than 1 mm.

**TABLE 1:** iView panel to isocenter plane measured distances in millimeters

Machine	iView panel location for panel to isocenter measurement (mm)				
	Gantry‐A	Target‐A	Gantry‐B	Target‐B	Center
A	588	593	589	595	592
B	589	594	590	595	593
C	593	598	593	598	595
D	590	595	589	596	593
E	580	581	581	580	579
F	585	586	586	587	586
G	583	586	584	587	585
H	589	594	590	595	593
I	590	594	590	594	591
J	598	601	598	601	600
Mean	588	592	589	593	591
St Dev	5	6	5	6	6
Range (Max–Min)	18	20	18	21	21

### 3.4 Inter‐machine variation

**FIGURE 7:**
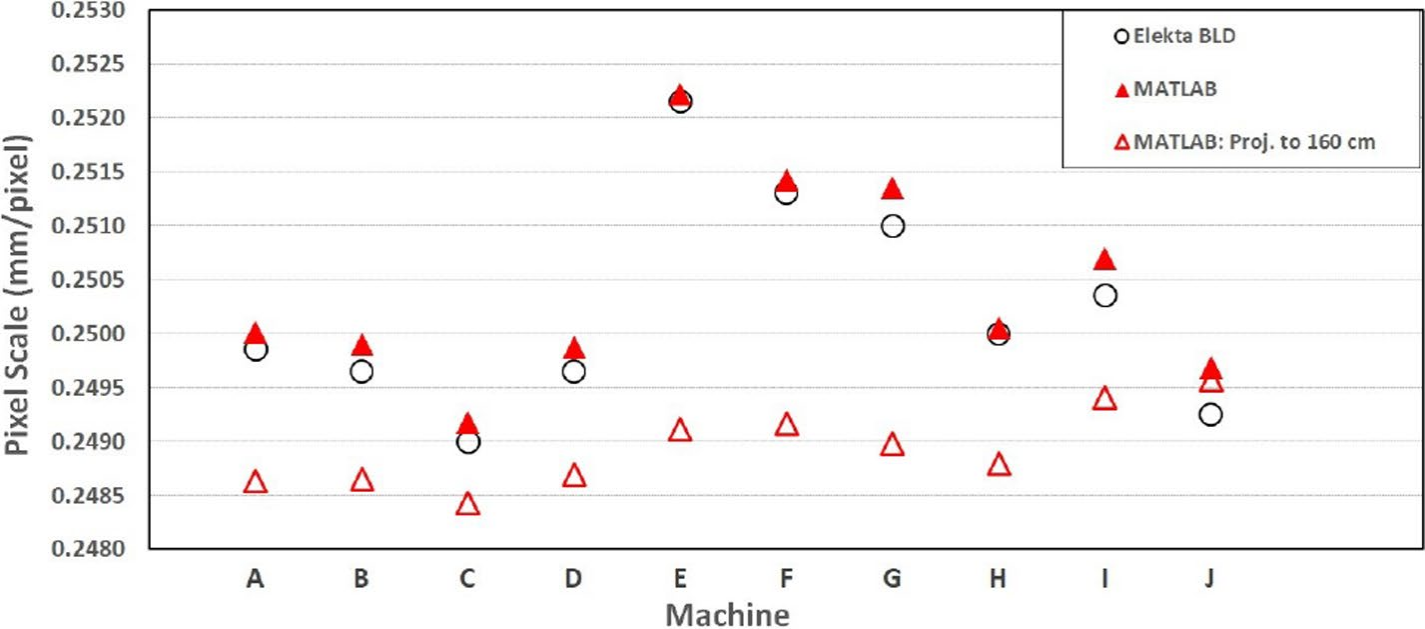
Pixel scale measured on 10 machines using calibration plate “A” calculated using the MATLAB script and the Elekta BLD workflow. MATLAB data is also shown normalized to 1600 mm source‐to‐panel distance (hollow triangles)

The averaged (cross‐plane and in‐plane) pixel scale factor across all 10 machines are plotted in Figure 7. Across all machines, MATLAB calculated pixel scale factors ranged between 0.2492 and 0.2522 mm/pixel, with a mean of 0.2504 (SD 0.001) mm/pixel. Mean difference of MATLAB relative to the Elekta BLD reference values was 0.0002 (SD 0.0001) mm/pixel with a maximum difference of 0.0004 mm/pixel on machine “J.”

To quantify the influence of machine source‐to‐panel variation, measured pixel scale factors were normalized to a nominal SPD of 1600 mm, as described by Equation (3). This data are presented in Figure 7. After correction, the range of calculated pixel scale factors reduced from 0.0030 to 0.0011 mm/pixel, with the mean changing from 0.2504 (SD 0.0010) mm/pixel to 0.2489 (SD 0.0004) mm/pixel.

(3) $$PixScale_{1600SPD}=PixScale_{\mathrm{meas}}\left(\frac{1000+IPD}{1600}\right)$$

## 4 DISCUSSION

Pixel calibration plate measured dimensions were within 0.1 mm of each‐other, which translated into a maximum 0.00012 mm/pixel difference where Vernier measured plate dimensions were used in place of nominal dimensions. This indicated the plates were inter‐changeable, and it was reasonable to use the nominal plate dimension as performed within the Elekta BLD workflow. As a component of routine equipment commissioning, it is still suggested that the dimensions should be checked on any new plate. The MATLAB calculated pixel scale factor measured slightly smaller (0.0001 mm/pixel) in the cross‐plane direction relative to the in‐plane for all plates. Measurements across all 10 machines (data not shown) gave analogous results with mean cross‐plane pixel scale factor being −0.0001 (SD 0.0001) mm/pixel relative to in‐plane. This could be due to manufacturing design of the Perkin Elmer panel, or due to panel sag, which was common across nearly all machines at the target end. Alternatively, this result may be due to the use of Elekta linear accelerators, which have been suggested to display a wider focal spot in the cross‐plane direction relative to in‐plane.^11^, ^12^ This would cause a wider cross‐plane beam penumbra, increasing the number of pixels measured, and a correspondingly smaller pixel scale factor which is consistent with the study findings.

Change in pixel scale factor with SSD followed a predictable linear trend, varying by 0.00025 mm/pixel for each millimeter error in SSD. The equipment used to calibrate and verify SSD such as mechanical front pointers and optical distance indicators typically have a resolution of 1 mm, however, even at a recommended tolerance of 2 mm defined by various publications uncertainty in pixel scale factor would be 0.0005 mm/pixel.^13^, ^14^ While small, it would still be recommended to verify the SSD with at least two independent methods (e.g., mechanical front pointer and optical distance indicator) prior to setting up the pixel plate.

The in‐house MATLAB script used in this study was shown to be in close agreement with pixel scale factor calculated from the Elekta BLD workflow. Reference Elekta BLD pixel scale factor data (measured over the preceding 12 months) closely matched the MATLAB calculated data across all machines. This demonstrates the stability of machine pixel scale factor over the long term. Furthermore, as the reference Elekta BLD data had been measured by different staff using different pixel plates, it demonstrates the insensitivity of pixel scale factor measurement due to staff, setup, or equipment uncertainties.

The type A uncertainties attributable to pixel plate dimension accuracy (±0.1 mm), SSD setup error (±1 mm), calculation algorithm (MATLAB calculated vs. Elekta BLD), and operator variation when added in quadrature sum to a pixel scale factor uncertainty of 0.0003 mm/pixel. This translates to an approximately 0.1 mm position error at 100 mm off axis in the isocenter plate. These various uncertainties investigated in this study were approximately an order of magnitude less than the variation seen across machines (0.003 mm/pixel). Therefore, none of these investigated uncertainties could be attributed to the inter‐machine variation observed.

The major contributing factor to inter‐machine pixel scale variation was source‐to‐panel distance. Some uncertainty may be attributed to definition of laser positioning, however, as laser calibration was performed using the same method on all machines, uncertainty in IPD due to laser position is estimated at less than 0.5 mm. For the Elekta Agility model, machine characteristics such as SPD are not easily modified, and therefore when using an EPID to define geometry at the isocenter plane (e.g., such as MLC calibration and QA), it is necessary to determine a unique machine/EPID pixel scale factor. Following removal or replacement of the iView panel, or following work on the support arm, it would be advised to re‐check the pixel scale.

Other factors not investigated in this paper which may explain the small residual inter‐machine variation include differences in beam focal spot size (i.e., effect on beam penumbra width), EPID manufacturing tolerances, and focal spot to isocenter distance.

## 5 CONCLUSION

The pixel scale factor is an important metric required for use of the EPID as a geometric tool when image distances are projected to isocenter. Inter‐machine variation in source‐to‐panel distance was shown to be the major factor influencing pixel scale, with all other setup, user, and plate manufacturing uncertainties being an order of magnitude less. It was shown that the pixel scale factor should be expected to vary across linacs of the same specifications. As a result, external software applications using Elekta Agility EPID images must have input of unique machine‐specific calibration factors if accurate geometric results are to be achieved at the isocenter plane.

## DATA AVAILABILITY STATEMENT

The data that support the findings of this study are available from the corresponding author upon reasonable request.

## CONFLICT OF INTEREST

No conflict of interest.

## AUTHOR CONTRIBUTION

This work (measurement, analysis, and write up) has been a collaboration between the authors listed, with Craig Norvill the lead author and Simon Goodall the second author.
